# Diagnostic and prognostic value of soluble CD14 subtype (Presepsin) for sepsis and community-acquired pneumonia in ICU patients

**DOI:** 10.1186/s13613-016-0160-6

**Published:** 2016-07-07

**Authors:** Kada Klouche, Jean Paul Cristol, Julie Devin, Vincent Gilles, Nils Kuster, Romaric Larcher, Laurent Amigues, Philippe Corne, Olivier Jonquet, Anne Marie Dupuy

**Affiliations:** Intensive Care Medicine Department, Lapeyronie University Hospital, 371, Av Doyen G. Giraud, 34295 Montpellier, France; PhyMedExp, INSERM U1046, CNRS UMR 9214, University of Montpellier, Montpellier, France; Department of Biochemistry, Lapeyronie University Hospital, Montpellier, France

**Keywords:** Severe sepsis, Septic shock, Diagnosis, Prognosis, Presepsin, Procalcitonin, Community-acquired pneumonia

## Abstract

**Background:**

The soluble CD14 subtype, Presepsin, appears to be an accurate sepsis diagnostic marker, but data from intensive care units (ICUs) are scarce. This study was conducted to evaluate the diagnostic and prognostic value of Presepsin in ICU patients with severe sepsis (SS), septic shock (SSh) and severe community-acquired pneumonia (sCAP).

**Methods:**

Presepsin and procalcitonin (PCT) levels were determined for patients at admission to ICU. Four groups have been differentiated: (1) absence or (2) presence of systemic inflammatory response syndrome, (3) SS or (4) SSh; and 2 groups, among the patients admitted for acute respiratory failure: absence or presence of sCAP. Biomarkers were tested for diagnosis of SS, SSh and sCAP and for prediction of ICU mortality.

**Results:**

One hundred and forty-four patients were included: 44 SS and 56 SSh. Plasma levels of Presepsin and PCT were significantly higher in septic than in non-septic patients and in SSh as compared to others. The sepsis diagnostic accuracy of Presepsin was not superior to that of PCT (AUC: 0.75 vs 0.80). In the 72/144 patients admitted for acute respiratory failure, the capability of Presepsin to diagnose sCAP was significantly better than PCT. Presepsin levels were also predictive of ICU mortality in sepsis and in sCAP patients.

**Conclusion:**

Plasma levels of Presepsin were useful for the diagnosis of SS, SSh and sCAP and may predict ICU mortality in these patients.

## Background

Despite advances in therapy, sepsis is the leading cause of death in critical care settings [1]. To improve the survival, early recognition of severe sepsis and septic shock and subsequent introduction of an aggressive supportive therapy are mandatory [2]. In routine clinical practice, early anti-infection treatment should be given before definitive diagnosis since blood culture, the gold-standard diagnostic method, usually takes several days to obtain the results and frequently yields low positive results. In fact, adequate microbiological information, ensuring appropriate therapy and avoiding unnecessary or unduly prolonged therapy, is lacking in more than 50 % of clinical situations. In this purpose, novel biomarkers have been developed and are being widely adopted in clinical settings. Among these biomarkers, procalcitonin (PCT) and high-sensitivity C-reactive protein (hs-CRP) are the main diagnostic markers used for bacterial sepsis. PCT is known to have the highest specificity, but its levels may increase in conditions without bacterial infection, such as severe trauma, invasive surgical procedure and critical burn injuries, thus resulting in false-positive results [3–5].

More recently, the soluble CD14 subtype, Presepsin, appears to be an accurate sepsis diagnostic marker and rises up a great clinical interest. Levels of Presepsin were found significantly higher in septic than in non-septic patients or those with SIRS [6]. Moreover, a specific increase was reported in the early stage of sepsis that also well correlated with severity [7]. Accordingly, plasma Presepsin levels could be useful for diagnosis and prognosis of sepsis and also for monitoring the course of the disease [8, 9]. Most of these studies have been, however, performed in settings of emergency departments [10–13], and data from intensive care units (ICUs) are scarce. Also, few studies have focused on community-acquired pneumonia [14–16]. In addition, plasma concentrations of Presepsin in most of previous reports were determined by ELISA method, which is time-consuming and not suitable for emergency. Yet, the new development of a fully automated point of care assay for rapid whole-blood Presepsin measurement updated its clinical use in emergency and ICUs [8, 11, 17].

Therefore, this study aimed to evaluate the diagnostic and prognostic utility of Presepsin measurements using the new fast method in severe sepsis and septic shock intensive care unit (ICU) patients. We also aimed to evaluate the diagnostic and prognostic utility of Presepsin measurements for severe community-acquired pneumonia (sCAP) in the subgroup of patients admitted to the ICU with acute respiratory failure.

## Methods

This observational prospective study was performed at 2 ICUs of Lapeyronie and Gui de Chauliac University hospitals of Montpellier, France. These two ICUs admit preferentially patients with suspected infectious diseases. It was carried out according to the principles of the Declaration of Helsinki and was approved by the Ethic Committee of Montpellier (Comité de protection des Personnes: CPP du CHU de Montpellier). Written informed consent was obtained from all participating patients or their closest relatives or legal representatives.

### Study population

All consecutive patients admitted to ICUs from January to May 2014 were included. Exclusion criteria were pregnancy, age < 18 years, previous congestive heart failure (class NYHA ≥ III), right ventricular failure, chronic renal failure stage III KDOQI or more, hepatic failure and acute pulmonary embolism.

### Methods

Baseline clinical variables including age, gender, cause of sepsis, and comorbidities were collected. The severity of disease was assessed by SAPS II [18] and SOFA scores [19]. At ICU admission, clinical and biological parameters including mean arterial pressure (MAP), serum creatinine, hsCRP, and PCT were also collected. ICU length of stay was recorded; ICU and in-hospital mortality were assessed.

Diagnosis of systemic inflammatory response syndrome (SIRS) and of sepsis severity was based on established criteria of the American College of Chest Physicians/Society of Critical Care Medicine [20]. Microbiological cultures were carried out. Patients who revealed a microbiologically or clinically proven infection were assigned to the sepsis group, and the others were considered as non-septic. Infection was considered clinically proven if the clinical picture and evolution complied with the diagnosis and if the patient was appropriately treated by antibiotics.

Community-acquired pneumonia (CAP) was defined as the presence of a new infiltrate on a chest radiograph and at least one of the following signs: cough, sputum production, dyspnea, core body temperature > 38.0 °C, auscultatory findings of abnormal breath sounds and rales [21]. Diagnosis may be confirmed by antigenuria or/and sputum cultures. Severe CAP (sCAP) was defined according to the American Thoracic Society guidelines [21].

Venous samples were taken from all patients at admission and immediately performed for Presepsin, PCT and hsCRP measurements. Presepsin concentration was measured by a chemiluminescent enzyme immunoassay (CLEIA) on a compact automatized immunoanalyzer PATHFAST^®^ (Mitsubishi Chemical Medience, Japan) recently evaluated [17]. The reference interval of the PATHFAST Presepsin assay determined from 127 healthy volunteers ranged from 92.7 to 398 pg/mL with an arithmetic mean of 189 pg/mL Presepsin values [17]. PCT was measured by commercial chemiluminescence assay on Kryptor^®^ immunoanalyzer (ThermoFisher, Agnières, France) following the manufacturers’ instructions. Determination of hsCRP was run on the Cobas8000/e502^®^ analyzer (Roche Diagnostic, Meylan, France) using immunoturbidimetric method.

Two study physicians (KK and VG) independently reviewed all available clinical, biological and radiological patients’ data and classified all patients into four disease groups: absence (non-SIRS) or presence of SIRS, severe sepsis (SS) or septic shock (SSh). The two study physicians followed recommended definitions and algorithms (20). Briefly, patients with SIRS and positive cultures were considered as septic. When cultures were non-contributive, clinical and biological picture (site of infection, clinical and biological picture and evolution), successful treatment by antibiotics and rule out of other diagnosis were main elements of sepsis diagnosis. Among the subgroup of patients who were admitted for acute respiratory failure, they reviewed also their data and classified them into two disease groups: absence or presence of sCAP (even in the absence of identified causative agent). When the study physicians cannot statute on the presence or not of sepsis, the patient was not included in the study. The study physicians and those on charge of patients were blinded to the results of Presepsin and PCT.

### Statistical analysis

The statistical analyses were performed using the STAT-VIEW II (Abacus Concepts Inc, Berkeley, CA). We first performed a descriptive analysis by computing the frequencies and the percents for categorical data, means, standard deviations, quartiles and extreme values for continuous data. We also checked for the normality of the continuous data distribution using the Shapiro–Wilks tests. We compared septic to non-septic patients and patients with and without sCAP for Presepsin, CRP and PCT measurements. The univariate analysis was performed using two-tailed Student’s *t* test, or two-tailed Mann–Whitney–Wilcoxon’s test when appropriate. Results were adjusted for multiple comparisons using Bonferroni’s method. Levels of significance for all tests were set at *p* < 0.05. Sensitivity, specificity and positive predictive value (PPV) and negative predictive value (NPV) of Presepsin and PCT for the diagnosis of sepsis and pneumonia were calculated using final diagnosis categorization based on clinical data, clinical scores and routinely used biomarkers levels. A receiver operating characteristic (ROC) analysis was performed for each of the biomarkers, and their diagnostic performance for sepsis and for other pathological condition was compared. The optimal threshold value was set for each ROC curve through the Youden Index (corresponding to the maximum of the sum “sensibility + specificity”). Mortality was displayed as Kaplan–Meier (log-rank test) plots according to the quartiles of Presepsin levels.

## Results

### Study population

**Fig. 1:**
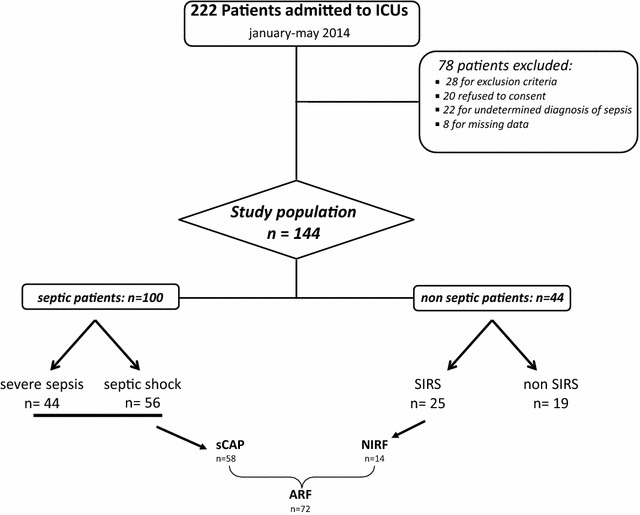
Flowchart for the study population. *SIRS* systemic inflammatory systemic response, *ARF* acute respiratory failure, *NIRF* non-infectious respiratory failure, *sCAP* severe community-acquired pneumonia

During the study period, a total of 222 critically ill patients were admitted in ICUs. After the exclusion of 78 patients, 144 were included: 88 males and 56 females. One hundred patients conformed to the criteria of bacterial sepsis: 44 with SS and 56 with SSh. Among the 44 non-septic patients, 19 were assigned for non-SIRS and 25 for SIRS. The screening process is shown in Fig. 1. The two study physicians were on total agreement on reviewing patient’s data (kappa = 1).

Patient’s baseline characteristics are summarized in Table 1. Non-septic and septic patients did not differ in age, sex, SAPS II score and current clinical and biological parameters, except for SOFA scores that were significantly higher in septic group. Forty of 100 septic patients experienced positive blood cultures. Severe pneumonia represented 58 % of sepsis causes (Table 2). Analyzing only the subgroup of patients (72) admitted for acute respiratory failure (ARF), sCAP was then diagnosed in 58 of them. Age and sex were not different between patients with infectious and non-infectious ARF, but SAPS II and SOFA scores were significantly higher in the infectious group (Table 3).

**Table 1:** Patient characteristics

	All patients *n* = 144	Non-sepsis *n* = 44	Sepsis *n* = 100	*p* value
Sex (male/female)	88/56	27/17	61/39	ns
Age, years (mean ± SD)	58 ± 17.5	57.5 ± 20.1	58.3 ± 16	0.907
SAPS II, median (IQR)	47 (33–62)	44 (27–60)	48 (36–64)	0.176
SOFA, median (IQR)	8 (6–11)	6 (4–10)	8 (6–11)	0.008
Creatininemia, median (IQR), (μmol/L)	68 (27–102)	80 (29–107)	57 (26–101)	0.419
hsCRP, median (IQR), (mg/L)	108 (38–133)	31 (5–87)	180 (81–284)	<0.0001
PCT, median (IQR), (ng/mL)	1.89 (0.32–13.7)	0.3 (0.1–0.9)	4.7 (0.8–20.5)	<0.0001
Presepsin, median (IQR), (pg/mL)	1058 (510–2090)	454 (315–915)	1432 (773–2337)	<0.0001
ICU length of stay (IQR), (days)	4 (2–10)	3 (1–7)	5 (2–11)	0.04
ICU mortality, *n* (%)	34 (23.6)	9 (20.4)	25 (25)	ns
In-hospital mortality, *n* (%)	38 (26.3)	10 (22.7)	28 (28)	ns

Comparison between septic and non-septic patients

*SAPS* simplified acute physiology score, *SOFA* sequential organ failure assessment score, *PCT* procalcitonin, *hsCRP* high-sensitivity C-reactive protein

*p*: differences between septic and non-septic patients

**Table 2:** Causes of infection in the 100 septic patients

Causes of infection	*n* 100
Pneumonia	58
Intra-abdominal infection	11
Meningitidis	8
Urinary infection	6
Isolated bacteremia	5
Others	6
Unknown	6

Forty patients had a positive blood cultures at ICU admission

**Table 3:** Characteristics of the subgroup of patients admitted for acute respiratory failure

	ARF 72	NIRF 14 (19.4 %)	Pneumonia 58 (80.5 %)	*p* value
Sex (male/female)	47/25	9/5	38/20	0.41
Age, years (mean ± SD)	61.7 ± 13.3	62.4 ± 13.4	61.6 ± 13.3	0.39
SAPS II, median (IQR)	47 (34–62)	37.5 (24–48)	48 (35–66)	0.01
SOFA, median (IQR)	8 (6–11)	5 (3–7)	9 (7–11)	0.0007
Creatininemia, median (IQR), (μmol/L)	60 (37–101)	86 (67–105)	50 (33–101)	0.22
Positive HAA, *n*	16	0	16	
hsCRP, median (IQR), (mg/L)	106 (51–245)	36 (23–105)	136 (71–270)	0.007
PCT, median (IQR), (ng/mL)	1.05 (0.28–8.84)	0.13 (0.09–0.34)	1.8 (0.3–11.4)	0.0006
Presepsin, median (IQR), (pg/mL)	989 (513–1951)	322 (231–534)	1209 (674–2195)	<0.0001
ICU LOS, median (IQR), (days)	5 (3–11)	4 (3–7)	6 (3–12)	0.21
ICU mortality, *n* (%)	15 (21)	1 (7)	14 (24)	0.01
In-hospital mortality, *n* (%)	18 (25)	2 (14.2)	16 (27.5)	0.04

Comparison between patients with infectious (pneumonia) and non-infectious respiratory failure (NIRF) at admission to ICU

*ARF* acute respiratory failure, *SAPS* simplified acute physiology score, *SOFA* sequential organ failure assessment score, *hsCRP* high-sensitivity C-reactive protein, *PCT* procalcitonin, *LOS* length of stay

*p*: differences between infectious and non-infectious respiratory failure patients

### Presepsin, PCT measurements

**Fig. 2:**
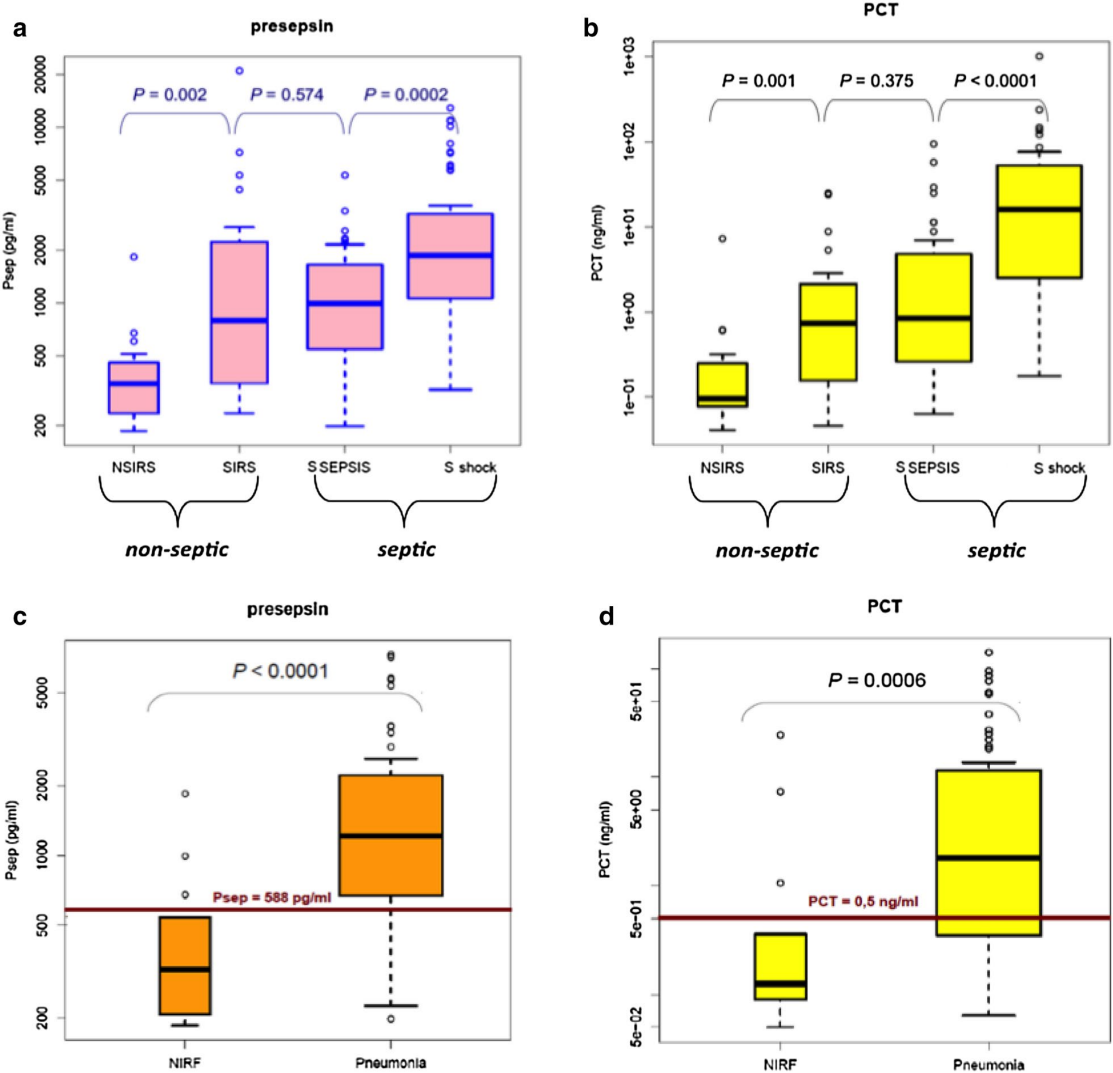
Comparison of Presepsin and PCT levels at ICU admission in all patients (**a**, **b**, respectively) and in the 72 patients with acute respiratory failure: infectious versus non-infectious origin (**c**, **d**, respectively). *SIRS* systemic inflammatory systemic response, *NSIRS* non-SIRS, *S Sepsis* severe sepsis, *S shock* septic shock, *NIRF* non-infectious respiratory failure

Significantly higher levels of hsCRP and PCT were found in septic as compared to non-septic patients (Table 1). Presepsin blood levels were also significantly more elevated in septic patients. Though Presepsin levels were significantly higher in septic as compared to non-septic patients, we observed non-significant differences in these levels between SIRS and severe sepsis groups (*p* = 0.574). In contrast, they were significantly higher in SSh versus SS and SIRS groups (Fig. 2a). Similar results were found regarding PCT levels (Fig. 2b). We extended our analysis to patients admitted for ARF and found that both Presepsin and PCT levels were significantly higher in patients with sCAP (Fig. 2c, d).

### Diagnostic accuracy and cutoff value of Presepsin

**Fig. 3:**
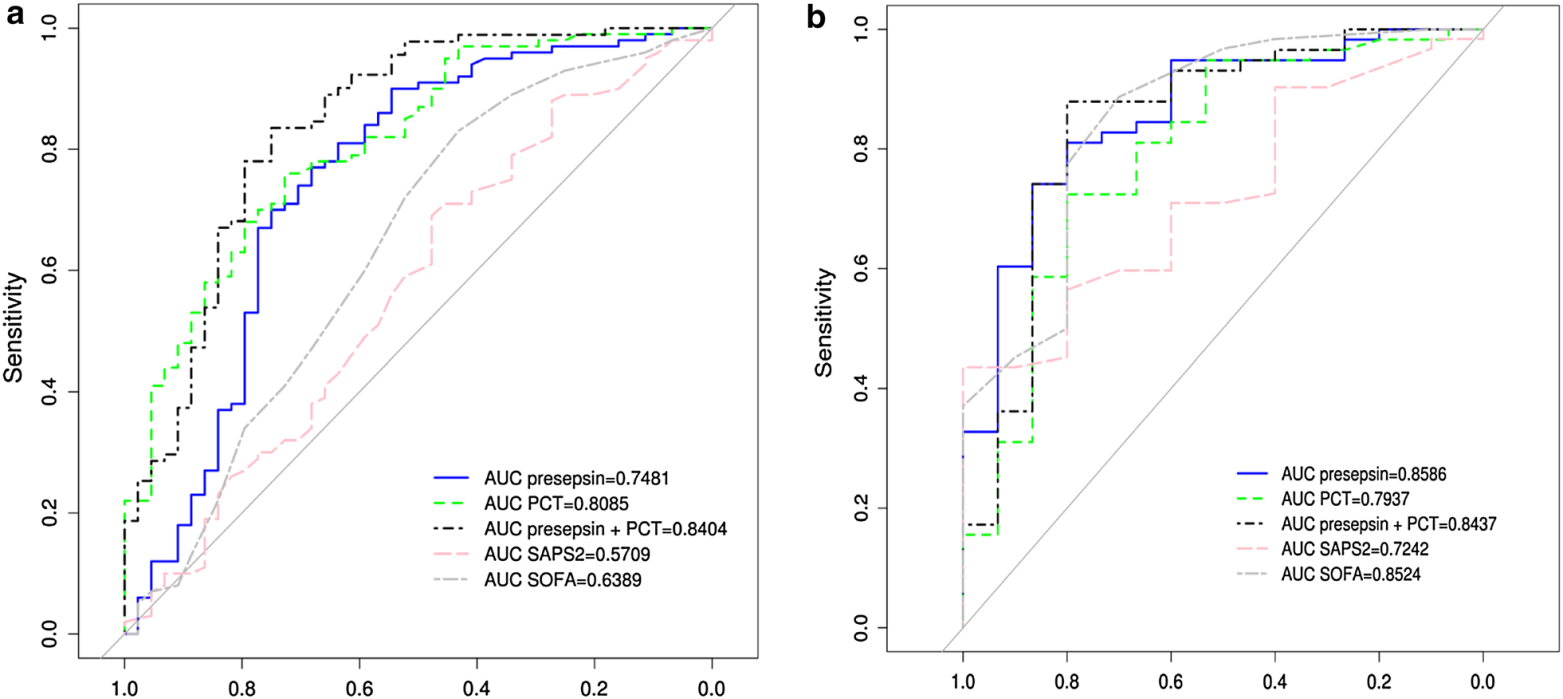
Receiver operating characteristic (ROC) for Presepsin, PCT, SAPS II and SOFA for diagnosis of sepsis (severe sepsis or septic shock) in all patients (**a**) and for diagnosis of pneumonia (infectious respiratory failure) in patients admitted for acute respiratory failure (**b**). *SAPS* simplified acute physiology score, *SOFA* sequential organ failure assessment score, *PCT* procalcitonin

The ROC curves were designed including those patients with a diagnosis of SS/SSh and are shown in Fig. 3a. The AUCs (areas under the curve) calculated from ROC curves were 0.75 for Presepsin and 0.80 for PCT, whereas those of SAPS II (0.57) and SOFA (0.64) were lower (Fig. 3a). When we combined Presepsin and PCT, AUC was at 0.84 (Fig. 3a). At a cutoff value of 466.5 pg/mL, sensitivity and specificity of Presepsin to severe sepsis and septic shock diagnosis were 90 and 55 %, respectively (Table 4). Lower sensitivity (80 %) and higher specificity (59 %) were observed for PCT (cutoff value: 0.5 pg/mL). The combination of Presepsin and PCT significantly improved specificity and PPV (Table 4).

**Table 4:** Sensitivity, specificity, positive predictive value (PPV) and negative predictive value (NPV) of PCT and Presepsin and their combinations for severe sepsis and septic shock and for pneumonia diagnoses

	Sensitivity (%)	Specificity (%)	PPV (%)	NPV (%)
SS and SSh				
PCT^a^	80	59	82	57
Presepsin^b^	90	55	82	71
PCT and Presepsin	75	68	85	55
Pneumonia				
PCT^a^	69	80	93	40
Presepsin^c^	81	80	94	52
PCT and Presepsin	62	93	97	62

*SS* severe sepsis, *SSh* septic shock, *PPV* positive predictive value, *NPV* negative predictive value

^a^Cutoff value for PCT at 0.5 ng/mL

^b^Cutoff value for Presepsin at 466 pg/mL

^c^Cutoff value for Presepsin at 588 pg/mL

The ROC curves were also designed including those patients admitted with ARF showed that the diagnostic value of Presepsin to discriminate infectious (sCAP) and non-infectious respiratory failure (AUC = 0.85) was higher than that of PCT (0.79), SAPS II (0.72), SOFA (0.78) scores, and similar to that of the combination of Presepsin and PCT (0.84) (Fig. 3b). Using a cutoff of Presepsin at 588 pg/mL, sensitivity (81 %), specificity (80 %), NPV and PPV values are greater than those of PCT (Table 4). The combination of Presepsin and PCT improved specificity, NPV and PPV reaching up to 97 %.

### Prognostic value of Presepsin levels

**Fig. 4:**
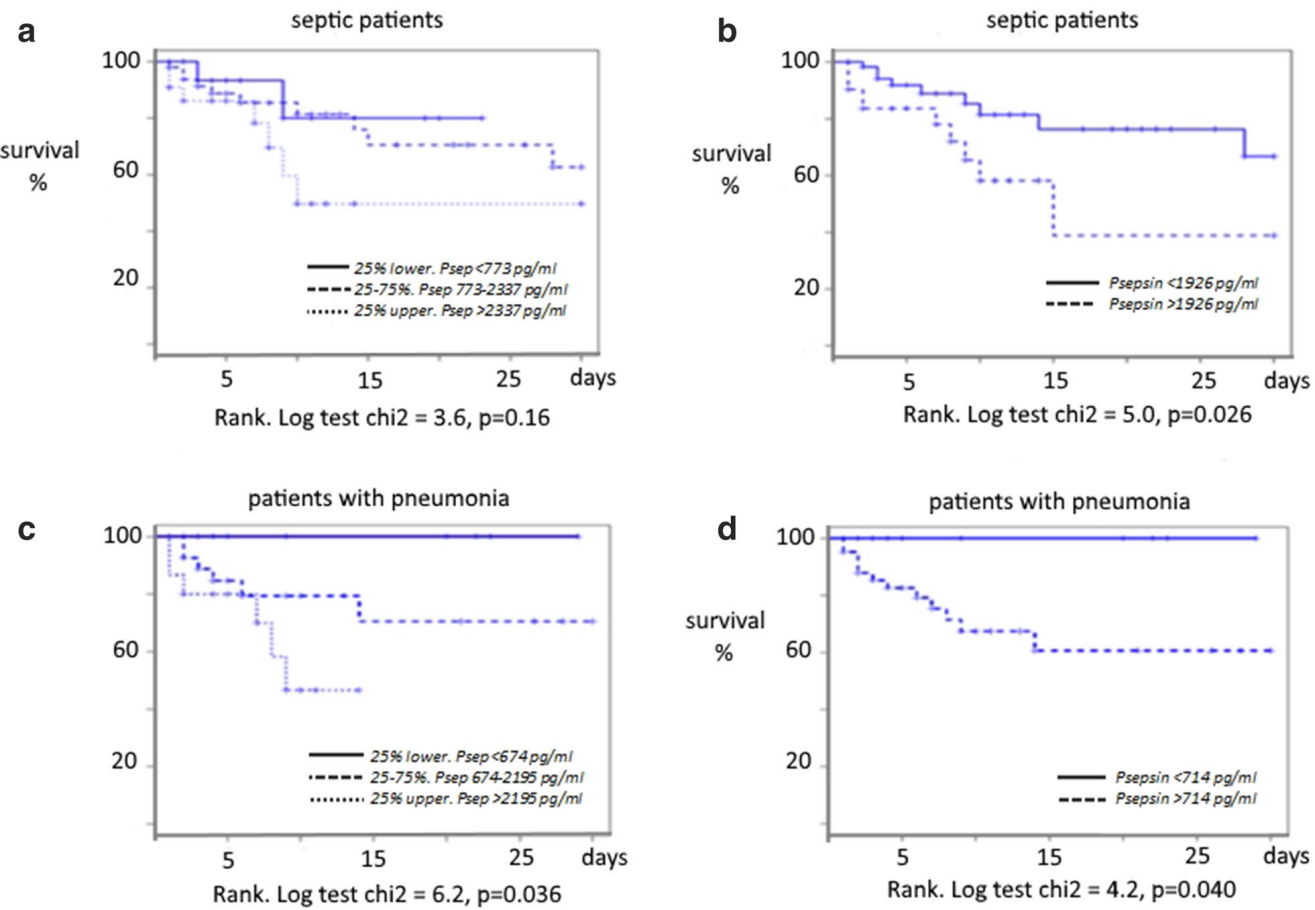
Kaplan–Meier graph showing correlation between plasma levels of Presepsin and survival in septic critically ill patients (**a**, **b**) and in critically ill patients with pneumonia (**c**, **d**). Survival curves according to the quartile of Presepsin (**a**) and to the cutoff of Presepsin (**b**) in septic critically ill patients, and in patients with pneumonia [according to the quartile of Presepsin (**c**) and to the cutoff of Presepsin (**d**)]. *p* values are given in each figure

Of the 100 septic patients included in the study, 25 (25 %) died during ICU stay. Deceased septic patients showed significantly higher Presepsin, PCT levels and severity scores at ICU admission (Table 5). After thirty ICU days, Kaplan–Meier curve assessing the impact of Presepsin levels on survival among critically ill septic patients did not show any differences according to the quartile of Presepsin levels (Fig. 4a). However, at a cutoff Presepsin value of 1926 pg/mL, mortality of septic patients was significantly higher in those with upper levels (Fig. 4b). Among the 58 patients with sCAP, 15 died at the ICU (mortality: 26 %). Plasma levels of Presepsin and PCT as well as SAPS II and SOFA scores were significantly higher in non-survivors patients (Table 6). Kaplan–Meier curves showed that patients with Presepsin of the upper quartile had significantly the highest mortality (Fig. 4c). The best cutoff value of Presepsin level to discriminate survivors from non-survivors was at 714 pg/mL (*p* = 0.04) (Fig. 4d).

**Table 5:** Comparison of clinical and biological variables at ICU admission between survivor and non-survivor septic patients

	Survivors 75	Non-survivors 25	*p* value
Sex (male/female)	43/32	18/7	0.06
Age, years (mean ± SD)	56.2 ± 19	64.6 ± 12	0.04
SAPS II, median (IQR)	41 (30–54)	65 (53–78)	<0.0001
SOFA, median (IQR)	7 (5–10)	10.5 (8–13)	<0.0001
Creatininemia, median (IQR), (μmol/L)	80 (32–103)	39 (20–68)	0.01
PCT, median (IQR), (ng/mL)	0.89 (0.20–11.4)	4.67 (1.89–24.8)	0.005
hsCRP, median (IQR), (mg/L)	95 (38–233)	150 (48–245)	0.24
Presepsin, median (IQR), (pg/mL)	871 (449–1828)	1734 (1014–3128)	0.0002
ICU length of stay, median (IQR), (days)	4 (2–11)	4 (2–10)	0.53

*SAPS* simplified acute physiology score, *SOFA* sequential organ failure assessment score, *PCT* procalcitonin, *hsCRP* high-sensitivity C-reactive protein

*p*: differences between survivor and non-survivor septic patients

**Table 6:** Comparison of clinical and biological variables at ICU admission between survivor and non-survivor patients with severe pneumonia

	Survivors 43	Non-survivors 15	*p* value
Sex (male/female)	28/15	8/7	
Age, years (mean ± SD)	62.4 ± 14	60.2 ± 10.9	0.22
SAPS II, median (IQR)	45(34–59)	66 (55–73)	<0.003
SOFA, median (IQR)	8 (6–11)	12 (9–13)	<0.005
Creatininemia, median (IQR), (μmol/L)	52 (33–100)	46 (30–95)	0.62
Positive HAA, *n*	11	4	
PCT, median (IQR), (ng/mL)	0.97 (0.27–8)	5.2 (2–16.8)	0.025
hsCRP, median (IQR), (mg/L)	102 (65–242)	180 (95–278)	0.29
Presepsin, median (IQR), (pg/mL)	999 (570–2020)	1821 (1310–2837)	0.006
ICU length of stay, median (IQR), (days)	6 (4–11)	5 (2–9)	0.36

*SAPS* simplified acute physiology score, *SOFA* sequential organ failure assessment score, *PCT* procalcitonin, *hsCRP* high-sensitivity C-reactive protein

*p*: differences between survivor and non-survivor patients

## Discussion

At ICU admission, plasma levels of Presepsin were found to be significantly higher in critically ill patients with sepsis in comparison with those without sepsis. Presepsin plasma levels of SIRS and SS patients were not significantly different, but patients with SSh had significant higher levels as compared to others. The sepsis diagnostic accuracy of Presepsin was not superior to that of PCT. With the combination of Presepsin and PCT, specificity and predictive positive value for sepsis were enhanced. We also demonstrated the usefulness of Presepsin for the diagnosis of sCAP in settings of ARF with an even better accuracy than PCT. Also, plasma Presepsin levels best predict ICU mortality in septic patients and those with sCAP at cutoff values of 1925 and 714 pg/mL, respectively.

It is now well demonstrated that sepsis, especially SS and SSh, should be diagnosed early and treated within 1 h after diagnosis [22]. Consequently, early sepsis biomarkers with a high sensitivity and specificity are required in addition to rapid detection methods. PCT, quickly measurable, is the most studied biomarker and is one allowing early diagnosis and management of therapy [4]. Presepsin is released, after intravenous administration of endotoxin in healthy patients, earlier than PCT [23–26], within the first 2 h. It reached a maximum after 3 h and returns to baseline concentrations after 4–8 h [27]. Indeed, higher blood levels of Presepsin were reported in infected patients as compared to non-infected and increased sequentially from SIRS, local infection to severe sepsis group [8]. In patients presenting to the emergency department, Liu et al. [10] demonstrated that Presepsin levels had the best capacity for diagnosis of sepsis at every stage, but Ulla et al. [13] reported a lower diagnostic value as compared to PCT. In this study, we enrolled exclusively critically ill patients admitted to ICU with potential concomitant organ failures and associated pathologies making differences between severe sepsis and SIRS inconsiderable. We found that Presepsin levels were not significantly different between SS and SIRS patients but significantly higher in SSh patients as compared to others. Median Presepsin concentrations at admission to the ICU of patients with SSh were at around 2000 pg/mL comparables to those reported by Liu et al. [10] and Carpio et al. [11] but differed from Ulla et al. [13] who reported higher values. Studying 116 severe sepsis and septic shock patients during their first week of ICU treatment, Behnes et al. [7] observed an increasing trend of Presepsin levels compared to controls from the lowest to the highest groups of sepsis severity. They also found, at day 1 of ICU treatment, that the value of Presepsin to diagnose septic shock (AUC = 0.80) was comparable to that of PCT (AUC = 0.83). The plotted ROC curves for our patients showed that the AUC for Presepsin was 0.75, less than PCT (0.80). In contrast, other studies showed that Presepsin has a better sensitivity and specificity in the diagnosis of sepsis than other biomarkers with an AUC at 0.845 (PCT: 0.65) [8]. Its sensitivity increased from 80.3 to 87.8 % and specificity from 78.5 to 81.4 % when the cutoff value was set from 399 to 600 pg/mL. In a multicenter study, the sensitivity of Presepsin for the diagnosis of sepsis was even higher at 91.9 %, significantly higher than PCT (89.9 %), IL6 (88.9 %) and blood cultures (35.4 %) [9]. A recent analysis of 246 patients admitted to the ICU reported a highest AUC of Presepsin at 0.948 but less than that of PCT (0.989) [28]. Presepsin cutoff levels to sepsis diagnosis varied from 400 to 600 pg/mL in emergency department settings but were above 500 pg/mL in ICU settings [7, 8]. At a diagnostic cutoff set at ≥466.5 pg/mL, we observed that Presepsin had a higher sensitivity (91 vs 80 %) and a lower specificity (55 vs 59 %) than PCT. Its diagnostic accuracy significantly improved when combined with PCT. Of note, a recent meta-analysis of accuracy of Presepsin for the diagnosis of sepsis included 8 studies investigating a total of 1815 patients (1165 sepsis and 525 SIRS) and showed that its AUC was 0.89 with a specificity at 78 % and a sensibility at 86 % but failed to determine the optimal cutoff value [16]. Presepsin was found suitable for the assessment of severity and prognosis of sepsis as well. At a cutoff value of 1925 pg/mL, its levels were predictive of ICU mortality in our septic patients. In the ALBIOS trial, a first analysis included 100 patients with SS and SSh, showing that median concentration of Presepsin at ICU admission was 2269 (1171–4300) pg/mL in deceased patients, which was significantly higher than 1184 (875–2113) pg/mL in survived [29]. Another study showed that Presepsin was better than IL6, CRP and PCT in assessing the risk of death within 30 days after onset of sepsis [10]. It was also shown that Presepsin had a valuable prognostic capacity to predict long-term all-cause mortality [7]. In addition, Presepsin levels were found to be correlated with APACHE II and SOFA scores [7] and to the appropriateness of antibiotherapy [29, 30]. Indeed, the second analysis from the ALBIOS trial (997 patients with severe sepsis or septic shock) demonstrated that Presepsin level was independently associated with the number and degree of organ dysfunctions or failures, coagulation disorders and ICU mortality [30].

More than half (58 %) of our septic patients have a sepsis from pulmonary origin. Diagnosis and severity of CAP are difficult and largely depend on the clinician’s experience since they are based on clinical and radiological arguments [31–33]. Circulating levels of PCT considered in the initial assessment of patients with signs and symptoms suggestive of CAP have a high predictive value in its clinical risk assessment and appear to be more specific for bacterial etiologies [34–36]. However, several observations suggest that PCT may vary with several factors including age, liver or renal dysfunction [36]. Some authors suggested that PCT should be regarded as a prognostic rather than a diagnostic factor [1, 37, 38]. Investigating more than 570 patients with CAP at the emergency department, Liu et al. [14] observed that Presepsin level was significantly higher in sCAP patients than in CAP patients and was predictive of mortality and that its combination with CURB65 score best predicted diagnosis and 28-day mortality. Our results extended these observations in ICU settings and demonstrated that Presepsin levels may be helpful in the identification of the infectious origin of an ARF. Moreover, the combination of Presepsin and PCT significantly improves their diagnostic value. Finally, we observed that Presepsin might also help to early stratify the risk and the prognosis of sCAP.

We must acknowledge some limitations to our study. First, our study was a bi-center study, and the results may not be directly applicable to all ICUs. Second, our population included a relative limited number of patients. Third, only plasma Presepsin levels at ICU admission were determined and dynamic and follow-up changes of this biomarker were not investigated [7]. Fourth, the diagnosis accuracy of Presepsin level may be affected by kidney function. Indeed, Nagata et al. [39] have shown an increase in Presepsin levels with decline in renal function but only for GFR below 30 mL/min. At the onset of Presepsin measurement, some of our patients have an impaired renal function but not a severe renal failure. Further studies should take into account renal function, especially if repetitive measurements are taken, knowing that Presepsin has a long half-life [40]. Fifth, in the analysis, we did not distinguish between patients with gram-negative and gram-positive bacterial infections due to the small number of patients. However, it is to note that sensitivity of Presepsin is not significantly different between gram-positive and gram-negative bacterial infections and even in case of fungal infection [9]. Lastly, we are aware that our results, although encouraging, may not already incite physicians to use this biomarker for sepsis diagnosis in ICUs. Obviously, further data and proofs are necessary to introduce a substantial change in our practice.

## Conclusion

Our results demonstrated the usefulness of Presepsin levels in the diagnosis and prognosis of septic shock patients admitted to ICUs, but its diagnostic ability remains moderate as recently demonstrated [41]. Its specificity and predictive positive value were enhanced with the association with PCT and should incite to evaluate their combination in further studies. The diagnostic accuracy of Presepsin in assessment of CAP was fair. Cutoff values of Presepsin for both diagnosis and prognosis of SSh and severe CAP have been determined herein but should be confirmed by further larger studies.
